# A model for predicting angiographically normal coronary arteries in survivors of out-of-hospital cardiac arrest

**DOI:** 10.1186/s40560-015-0099-y

**Published:** 2015-07-15

**Authors:** Toshikazu Abe, Shigeyuki Watanabe, Atsushi Mizuno, Masahiro Toyama, Vicken Y. Totten, Yasuharu Tokuda

**Affiliations:** Department of Emergency and Critical Care Medicine, Tsukuba Medical Center Hospital, 1-3-1, Amakubo, Tsukuba, Ibaraki 305-0005 Japan; Department of Cardiology, Mito Kyodo General Hospital, University of Tsukuba, Tsukuba, Ibaraki Japan; Department of Cardiology, St. Luke’s International Hospital, Tokyo, Japan; Kaweah Delta Health Care District, Visalia, CA USA; Japan Community Healthcare Organization, Tokyo, Japan

**Keywords:** Out-of-hospital cardiac arrest, Acute coronary syndrome, Coronary angiography, Electrocardiogram, Post-cardiac arrest care

## Abstract

**Background:**

It has been recommended that all survivors of out-of-hospital cardiac arrest (OHCA) have immediate coronary angiography (CAG), even though it has been reported that half of the survivors have normal coronary arteries. Our aim was to develop a model which might identify those who have angiographically normal coronary arteries. Reliable prediction would reduce unnecessary CAG.

**Methods:**

A retrospective, observational, cohort study was conducted on 47 consecutive adult survivors who received immediate CAG after resuscitation from OHCA, between June 1, 2006 and March 31, 2011. We analyzed the clinical and electrocardiographic characteristics of the survivors with and without normal coronary arteries.

**Results:**

All subjects had CAG. Normal coronary arteries were found in 25/47. These persons did not have diabetes mellitus (*p* = 0.0069) or a history of acute coronary syndrome (ACS) (*p* = 0.0069). Any abnormality of the ST segment or ST segment elevation on electrocardiogram (ECG) was strongly related to abnormal coronary arteries (*p* = 0.0045 and *p* = 0.0200, respectively). The partitioning model for predicting angiographically normal coronary arteries showed that all patients (8/8) with no ST segment change on their ECG had normal coronary arteries. Eight out of ten patients with ST segment abnormalities also had normal coronary arteries with a history of arrhythmia without a history of ACS.

**Conclusions:**

Survivors of OHCA who have no history of diabetes mellitus, who have no past history of ACS, and who present with no ST segment abnormalities may not require urgent/emergent CAG. Further studies are needed to guide clinicians in the determination of emergent cardiac catheterization following resuscitation of OHCA.

## Background

The most common causes of cardiac arrest are cardiovascular disease and coronary ischemia [1, 2]. The American Heart Association (AHA)’s Advanced Cardiovascular Life Support (ACLS) guidelines recommend that a 12-lead electrocardiogram (ECG) should be obtained as soon as possible after resuscitation from cardiac arrest, to find potentially treatable acute coronary syndrome (ACS). ST elevation, new (or presumably new) left bundle branch block suggests that cardiac arrest was due to ACS [3]. However, cardiac catheterization is commonly performed on all survivors of out-of-hospital cardiac arrest (OHCA) even in the absence of ST elevation [1, 4, 5]. Since chest pain and ST segment elevation have been shown to be poor predictors of ACS, immediate coronary angiography (CAG) has been recommended even in the absence of ST segment elevation because of the high incidence of ACS among the survivors [1, 4]. Additionally, comatose cardiac arrest patients cannot give a history. Since their post-resuscitation ECG findings and history are unreliable, Neumar et al. recommended that all comatose cardiac arrest survivors should have CAG and percutaneous coronary intervention (PCI) [6]. Therefore, CAG is commonly performed if patients seem to have a chance of a neurologically favorable outcome. Among patients with OHCA admitted to intensive care for hypothermia, Nielsen et al. reported that half of the patients had CAG and one-third had PCI [7]. Yet, the Spaulding study suggests that half of the patients who received CAG might not have needed it [1].

CAG is invasive and expensive and carries a certain morbidity and mortality. Both CAG and the contrast agents are potentially harmful. Survivors of OHCA may be unstable. Delayed invasive procedures may be safer. Therefore, it is important to identify which OHCA patients have angiographically normal coronary arteries and for whom delay in CAG is safe.

## Methods

### Ethics statement

The study protocol was reviewed and approved by the ethics committee of Mito Kyodo General Hospital, University of Tsukuba Hospital Mito Medical Center. The ethics committee at our institution does not require its approval for observational studies using anonymous data in existence such as this study. Also, informed consent from each patient was waived for using anonymous data according to the informed consent guidelines in Japan.

A retrospective, observational, cohort study was conducted on consecutive adult patients (age ≥18 years) who were survivors of OHCA and who received immediate CAG. These patients all presented to the emergency department (ED) of an urban teaching hospital in Japan, between June 1, 2006 and March 31, 2011. The ED of St Luke’s International Hospital, Tokyo, provides primary to tertiary care to a population of approximately 100,000. The management of OHCA involves the Tokyo Fire Department (TFD) and the EDs of other hospitals in Tokyo. Typically, the closest emergency medical technicians (EMTs) are dispatched to the scene. Cardiopulmonary resuscitation (CPR) is initiated by EMTs at arrival and continued according to the AHA standards. A 12-lead ECG is performed in the ED immediately after return of spontaneous circulation (ROSC).

ROSC patients were brought directly from the ED to the cardiac catheterization laboratory. CAG was performed according to a standard technique. Experienced cardiologists made the decision to proceed to angioplasty only for critical lesions. Standard resuscitation and stabilization were used during and after the procedure.

### Data collection

The data were retrospectively obtained from computerized medical records and collected in the Utstein style. Variables included age, gender, height, weight, risk factors of ACS including hypertension (HT), hyperlipidemia (HL), diabetes mellitus (DM), history of ACS, PCI, coronary artery bypass graft (CABG), heart failure, arrhythmia, chest pain before arrest, witnessed collapsed patient, bystander initiated CPR, ventricular tachycardia/ventricular fibrillation (VT/VF) on EMT arrival, estimated time of initiation of CPR, and estimated time of cardiac arrest (interval until ROSC). The primary outcomes were the CAG findings, (including normal coronary artery or not). Secondary outcomes included PCI results intra-aortic balloon pumping (IABP), venoarterial-extracorporeal membrane oxygenation (VA-ECMO), and therapeutic mild hypothermia. Normal coronary arteries were defined as ‘no stenosis of any coronary arteries’ by experienced cardiologists’ reading.

Patients’ prognoses 1 month after admission were categorized by the Glasgow-Pittsburgh cerebral performance category (GP-CPC) scale, from category 1 (good cerebral performance), category 2 (moderate cerebral disability), category 3 (severe cerebral disability), category 4 (coma or vegetative state), to category 5 (death). We also obtained data on survival 1 month after resuscitation.

All ECG were recorded just after ROSC at the ED. ECGs were interpreted by two experienced cardiologists who were unaware of the patients’ angiographic status. Disagreement between the two experienced cardiologists was arbitrated by an independent third party. ECG findings recorded included the following: heart rate (HR), axis deviation, atrial fibrillation (AF) or atrial flutter (AFL), junctional rhythm, presence or absence of P wave, abnormal shape of P wave, abnormal PQ interval, prolonged PQ interval, any abnormal QRS shape, wide QRS, right bundle branch block (RBBB), left bundle branch block (LBBB) including left anterior hemiblock (LAH) or left posterior hemiblock (LPH), any bundle branch block (BBB), bifascicular block, presence or absence of Q wave, any abnormal ST segment change, ST segment elevation, ST segment depression without reciprocal change, any ST segment depression, prolonged QT interval, presence or absence of inverted T wave (negative T wave, coronary T wave, or flat T wave), and abnormal U wave. Abnormal ST depression is defined as ST depression of >1 mm (0.1 mV) measured at 80 ms after the J point in at least two contiguous leads. Abnormal ST elevation is defined as ST elevation at the J point in at least two contiguous leads of >2 mm (0.2 mV) in men or >1.5 mm (0.15 mV) in women in leads V2–V3 and/or of >1 mm (0.1 mV) in other contiguous chest leads or the limb leads. They were also asked to comment on whether patients might have ACS based on the ECG findings.

### Selection of participants

During the study period, there were 1390 patients with OHCA, of whom 472 patients (34 %) were admitted to our hospital. Angiography was performed on 49 patients. Criteria for CAG were as follows: probable favorable neurological outcome and no obvious non-cardiac causes for the arrest, such as hyperkalemia, intoxication, or trauma. Two CAG patients were excluded because of missing ECG data, leaving 47 patient cases analyzed. The reason why we decided the patient would have probable favorable neurological outcome was that most participants had witnessed cardiac arrest or collapse, bystander initiated CPR, and a relatively short duration prior to ROSC.

### Statistical analysis

The primary outcome was ‘normal coronary arteries’ according to CAG evaluation. Predictor variables were assessed by univariate analysis with Fisher’s exact test for categorical variables and *t* test for continuous variables. A two-tailed *p* value of less than 0.05 was considered statistically significant. We also analyzed the inter-rater reliability of the cardiologist’s assessment of ECGs using the kappa coefficient. Based on univariate analysis, these five variables were chosen as the predictor (independent) variables: age, history of ACS, history of arrhythmia, history of DM, or any abnormal ST segment change on the ECG. All patients 50 years old and younger had normal coronary arteries. Although age was one of the strongest predictors of normal coronaries, it is difficult to generalize this predictor to other populations because Japanese under 50 are at relatively low risk of ACS compared with people in other countries, so it was not used as a generalizable variable.

A multiple logistic regression model could not identify significant factors of the ECG findings because all patients with no abnormal ST change on ECG had cardiac arrest with normal coronary arteries. Thus, a recursive partitioning model was fit by analyzing the relationship between cardiac arrest with normal coronary arteries and the chosen four predictors. The recursive partitioning was conducted by maximizing the entropy index. Validation is the process of using 90 % of this data set to estimate model parameters and using the 10 % part to assess the predictive ability of the model using k-fold cross-validation. Sensitivity analysis of the model was performed using the area under the curve (AUC). Inter-rater reliability was analyzed by STATA version 11.2 (StataCorp, Texas). The other analyses were performed with JMP version 9.0.3 (SAS Institute, Cary, NC).

## Results

The main clinical and preadmission characteristics of the 47 patients are shown in Table 1. The mean age was 55.4 ± 15.9. The males were 42/47 (89.4 %). Thirty-seven out of forty-seven (78.7 %) patients had witnessed collapsed. Thirty-three out of forty-seven (70.2 %) patients received bystander CPR. Forty-four out of forty-seven (93.6 %) patients had VT/VF recognized on EMTs arrival. The median interval to ROSC was 15 min (Q1–Q3 10–22 min).

**Table 1:** Clinical and preadmission characteristics of 47 patients

Characteristics			Value	
Age (mean ± SD)			55.4 ± 15.9	
Male (*n* (%))			42 (89.4)	
Height (mean ± SD)			167.6 ± 8.5	
Weight (mean ± SD)			64.4 ± 11.3	
Past history (*n* (%))	HT		17 (36.2)	
	HL		7 (14.9)	
	DM		6 (12.8)	
	ACS		6 (12.8)	
	PCI		5 (10.6)	
	CABG		3 (6.4)	
	Heart failure		6 (12.8)	
	Arrhythmia		13 (27.8)	
Chest pain before arrest			5 (10.6)	
Witness			37 (78.7)	
Bystander initiated CPR			33 (70.2)	
VT/VF on EMT arrival			44 (93.6)	
Median interval of initiation of CPR (min (Q1–Q3))			4 (1–7)	
Median interval of ROSC (min (Q1–Q3))			15(10–22)	

*HT* hypertension, *HL* hyperlipidemia, *DM* diabetes mellitus, *ACS* acute coronary syndrome, *PCI* percutaneous coronary intervention, *CABG* coronary artery bypass graft, *CPR* cardiopulmonary resuscitation, *VT/VF* ventricular tachycardia/ventricular fibrillation, *EMT* emergency medical technician, *ROSC* return of spontaneous circulation

Q1 25 % interquartile, Q3 75 % interquartile

According to CAG, 25/47 (53 %) patients had normal coronary arteries and 22 (47 %) patients had abnormal coronary arteries. In the 22 patients with abnormal coronary arteries, 19 patients underwent PCI. Three patients did not receive PCI. One patient had 50 % stenosis of segment # 11 coronary artery, and one patient had 75 % stenosis of # 3 coronary artery. However, the stenoses of their coronary arteries were rapidly dilated by administering isosorbide dinitrate. One patient had known 100 % stenosis of # 7 coronary artery, with akinesis of the apex by left ventriculogram (LVG), which was not amenable to stenting. Nine out of forty-seven (19 %) patients received IABP, and two out of forty-seven (4 %) patients received VA-ECMO. Forty out of forty-seven (85 %) patients underwent therapeutic mild hypothermia.

All patients had survived 1 month after admission. At that time, the neurologic status of 45/47 (96 %) patients was GP-CPC 1, one patient was GP-CPC 2, and one was GP-CPC 3. There was no patient with GP-CPC 4 or 5 (Table 2).

**Table 2:** Therapeutic intervention and prognosis among survivors

		Number	Percent
CAG			
Normal coronary		25	53
PCI		19	40
IABP		9	19
VA-ECMO		2	4
Therapeutic mild hypothermia		40	85
GP-CPC	1	45	96
	2	1	2
	3	1	2
	4	0	0
	5	0	0
1-month survival		47	100

*CAG* coronary angiography, *PCI* percutaneous coronary intervention, *IABP* intra-aortic balloon pumping, *VA-ECMO* venoarterial-extracorporeal membrane oxygenation, *GP-CPC* Glasgow Pittsburgh cerebral performance category

Table 3 shows the univariate analysis of the patients’ demographics and ECG findings compared with the status of coronary arteries. Age younger than 50 was related to normal coronary arteries (*p* < 0.001). No one with normal coronary arteries had a past history of DM, ACS, or PCI (*p* = 0.0069, *p* = 0.0069, *p* = 0.017, respectively). VT/VF on EMT arrival was not related to abnormal coronary arteries (*p* = 1.0000). Chest pain before arrest was not related to status of coronary arteries (*p* = 1.0000). Any abnormal ST segment change (i.e., an integrated finding of ST segment elevation or depression) or ST segment elevation on ECG was related to abnormal coronary arteries, respectively (*p* = 0.0045, *p* = 0.0200). ST segment depression only was not statistically related to status of coronary arteries. Expert opinions relatively accurately predicted the presence of normal coronary arteries. Inter-rater reliability of experts opinions was intermediate (*κ* = 0.5616).

**Table 3:** Univariate analysis of patients’ demographics and ECG findings compared with status of coronary arteries

		Unit	Abnormal coronary (*n* = 22)	Normal coronary (*n* = 25)	*p* value
	Age	Mean ± SD	65 ± 9	47 ± 16	<0.001
	Gender (Male)	*n* (%)	22 (100)	20 (80)	0.0518
Past history	HT	*n* (%)	9 (41)	8 (32)	0.5583
	HL	*n* (%)	4 (18)	3 (12)	0.6902
	DM	*n* (%)	6 (27)	0 (0)	0.0069
	ACS	*n* (%)	6 (27)	0 (0)	0.0069
	PCI	*n* (%)	5 (23)	0 (0)	0.0172
	CABG	*n* (%)	3 (14)	0 (0)	0.0950
	Heart failure	*n* (%)	2 (9)	4 (16)	0.6701
	Arrhythmia	*n* (%)	3 (14)	10 (40)	0.0561
Prehospital status	Chest pain before arrest	*n* (%)	2 (9)	2 (8)	1.0000
	VT/VF on EMT arrival	*n* (%)	21	23	1.0000
ECG findings	HR	Mean ± SD	96 ± 29	84 ± 23	0.1393
	Axis deviation (RAD or LAD)	*n* (%)	0 (0)	3 (12)	0.8368
	AF or AFL	*n* (%)	7 (32)	6 (24)	0.7450
	Junctional rhythm	*n* (%)	1 (5)	3 (12)	0.6115
	P wave	*n* (%)	14 (64)	21 (84)	0.1800
	Abnormal P wave	*n* (%)	7 (32)	4 (16)	0.3027
	Abnormal PQ interval	*n* (%)	9 (410)	9 (36)	0.7712
	Prolonged PQ	*n* (%)	1 (5)	5 (20)	0.1936
	Any abnormal QRS	*n* (%)	16 (73)	19 (76)	1.0000
	Wide QRS	*n* (%)	1 (5)	3 (12)	0.6115
	QRS width (ms)	Mean ± SD	98 ± 32	91 ± 25	0.4028
	RBBB	*n* (%)	6 (27)	6 (24)	1.0000
	LBBB (LAH or LPH)	*n* (%)	1 (5)	4 (16)	0.3525
	Any BBB	*n* (%)	7 (32)	8 (32)	1.0000
	Bifascicular block	*n* (%)	1 (5)	2 (8)	1.0000
	Q wave	*n* (%)	12 (55)	9 (36)	0.2481
	Any abnormal ST segment change	*n* (%)	22 (100)	17 (68)	0.0045
	ST segment elevation	*n* (%)	14 (64)	7 (28)	0.0200
	ST segment depression without reciprocal change	*n* (%)	7 (32)	10 (40)	0.7617
	Any ST segment depression	*n* (%)	16 (73)	13 (52)	0.2293
	Prolonged QT interval	*n* (%)	12 (55)	14 (56)	1.0000
	Invert (coronary or negative or flat)	*n* (%)	5 (23)	1 (4)	0.0848
	Abnormal U wave	*n* (%)	4 (18)	4 (16)	1.0000
Expert opinions^a^	Cardiologist # 1 assessment of ECG	*n* (%)	16 (73)	20 (80)	0.0004
	Cardiologist # 2 assessment of ECG	*n* (%)	16 (59)	21 (84)	0.0029

*ECG* electrocardiogram, *HT* hypertension, *HL* hyperlipidemia, *DM* diabetes mellitus, *ACS* acute coronary syndrome, *PCI* percutaneous coronary intervention, *CABG* coronary artery bypass graft, *CPR* cardiopulmonary resuscitation, *VT/VF* ventricular tachycardia/ventricular fibrillation, *EMT* emergency medical technician, *HR* heart rate, *RAD* or *LAD* right axis deviation or left axis deviation, *AF* or *AFL* atrial fibrillation or atrial flutter, *RBBB* right bundle branch block, *LBBB* left bundle branch block, *LAH* left anterior hemiblock, *LPH* left posterior hemiblock, *BBB* bundle branch block

^a^ *K* = 0.5616

**Fig. 1:**
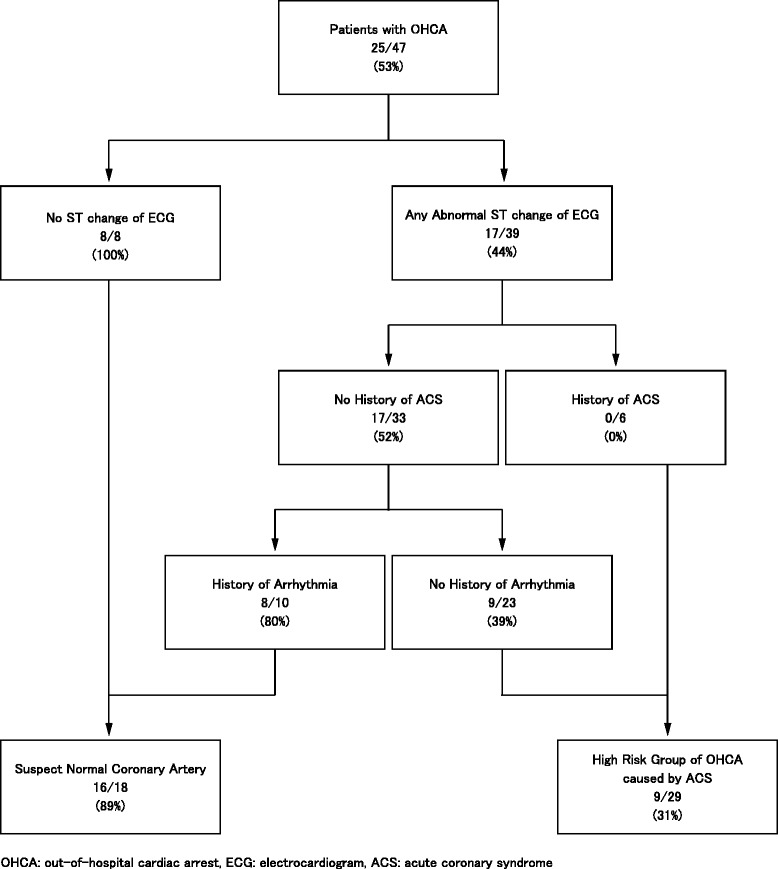
The partitioning model for predicting angiographically normal coronary arteries among survivors from OHCA

The partitioning model for predicting angiographically normal coronary arteries (Fig. 1) showed that all patients (8/8) with no ST segment change on their ECG at the ED had normal coronary arteries. Also, 8/10 (80 %) patients had normal coronary arteries if they had no history of ACS and when they had history of arrhythmia even if some ST segment changes were shown on their ECGs. Those were the low-risk group with OHCA caused by ACS, which indicates they had normal coronary arteries. On the other hand, all patients (6/6) had abnormal coronary arteries when any ST segment changes were shown on their ECGs and they had a past history of ACS. When patients had any abnormal ST segment changes on their ECG, no history of ACS, and no history of arrhythmia, 14/23 (61 %) patients had abnormal coronary arteries. Those nodes were the high-risk group of cardiac arrest caused by ACS (abnormal coronary arteries). The AUC of this model was 0.8004.

## Discussion

We showed that a simple model may predict normal coronary arteries in OHCA patients. Survivors of OHCA may not need immediate CAG if no ST segment changes are shown on their ECG at ED, or if some ST segment changes are shown when they have no history of ACS but they have a history of arrhythmia.

CAG was used as the gold standard for AMI definition, as immediate CAG was performed after resuscitation in all patients, irrespective of the ECG changes. The proportion of patients with abnormal coronary arteries in our study population was similar to that of the previous study [1]. It is slightly different from that of another study [7]. The reason why proportion of abnormal coronary arteries was slightly different is the definition of normal coronary arteries was slightly different between our study and prior studies. However, it did not become a major issue for our purpose because our definition of normal coronary arteries was most strict.

The mean age was relatively younger compared to the mean age of other Japanese studies of CPR [8, 9]. Our results confirmed that younger age was a predictor for a favorable outcome. One explanation for the preponderance of males in our sample may be that our hospital is in a business area in a big city; however, the gender ratio is similar to other studies [10].

Although the previous study reported that ST segment elevation or depression were poor predictors of acute coronary artery occlusion [1, 11], we investigated the relationship between ECG findings and normal coronary arteries instead of abnormal coronary arteries (e.g. ACS) because our aim was to reduce unnecessary CAG for critical patients beyond the current criteria. It is the advantage of our study compared with other studies [1, 2, 10–13]. The ability of a normal ECG to predict normal arteries is greater than the ability of an abnormal ECG predicting abnormal arteries. ECG change itself demonstrates to predict normal coronary arteries more precisely than abnormal ones in clinical practice because it shows not only ischemia but also secondary results of cardiac arrest or resuscitation procedures [11]. In addition, expert opinions could also more precisely predict normal coronary arteries than abnormal ones in our results. Some studies reported ST segment elevation was strongly related to ACS (as in our results) although a finding of ‘no ST segment elevation’ did not result in postponing CAG in their study [2, 12, 13]. We also found that any vertical movements of the ST segment of the ECG were related to the status of the coronary arteries, although ST segment elevation was more important than its depression. Sideris et al. said that combined ECG criteria (either upward or downward movement) might improve the diagnostic value of the ECG. They found a negative predictive value (NPV) of 100 % by using combined ECG criteria [10]. Their study used a design similar to ours but used more complicated criteria. It seems that our model could predict normal coronary arteries better than abnormal ones. Moreover, compared to other studies, our results are similar to actual clinical practice because of the partitioning model [10, 11].

McMullan et al. described the reluctance of interventional cardiologists to routinely perform PCI in such high-risk patients [14]. PCI-related death included death post PCI, and ROSC is publicly reported as a measure of quality [15].

Some survivors of OHCA are low risk (18/47: 38 % of all patients) and did not need immediate CAG. This does not imply that the group at high risk for coronary artery occlusion should not receive immediate CAG.

### Limitations

Our study has limitation. First, our study population was limited to the 10 % of all patients who were admitted after OHCA, the ones who had immediate CAG because they were expected to have a good neurologic outcome. This might have introduced a selection bias; the decision to go to CAG was made according to the experience of physicians. Other previous studies also suffered from the same limitations [10, 11]. Fortunately, the demographics of our patients were similar to those of previous studies, making the studies more comparable [1, 7, 10]. It is not difficult for physicians to clinically select patients expected to have a poor neurological outcome. For this study, our aim was to further reduce CAG by creating selection criteria associated with normal coronary arteries [10]. Our algorithm predicted normal coronary arteries by ECG and history. It is possible that the arteries were only “angiographically” normal but might have ACS due to coronary vasospasm [16, 17], or oxygen demand and supply imbalance due to unclear pathophysiology [17]. We doubt this, however, because Japanese cardiologists usually additionally investigate the coronary arteries with the use of intravascular ultrasound (IVUS), acetylcholine provocation test, and thrombectomy if the patient’s status allows it. Thus, we think our results actually showed normal coronary arteries; those with ACS and normal arteries also do not need immediate CAG just after ROSC. Further study, including external validation, is needed to overcome these limitations.

## Conclusions

This was a retrospective review of OHCA survivors to determine if certain features—the absence of ST segment deviation on the ECG—and minimal ST segment deviation yet lacking a past history of ACS and having a past history of arrhythmia would predict angiographically normal arteries. If substantiated, CAG could be postponed in patients who meet these criteria. Because of the small sample size and retrospective design, the findings are not yet robust enough to suggest changes to current protocols.

## References

[1] Spaulding CM, Joly LM, Rosenberg A, Monchi M, Weber SN, Dhainaut JF (1997). Immediate coronary angiography in survivors of out-of-hospital cardiac arrest. N Engl J Med.

[2] Anyfantakis ZA, Baron G, Aubry P, Himbert D, Feldman LJ, Juliard JM (2009). Acute coronary angiographic findings in survivors of out-of-hospital cardiac arrest. Am Heart J.

[3] Peberdy MA, Callaway CW, Neumar RW, Geocadin RG, Zimmerman JL, Donnino M (2010). Part 9: post-cardiac arrest care: 2010 American Heart Association Guidelines for Cardiopulmonary Resuscitation and Emergency Cardiovascular Care. Circulation.

[4] Reynolds JC, Callaway CW, El Khoudary SR, Moore CG, Alvarez RJ, Rittenberger JC (2009). Coronary angiography predicts improved outcome following cardiac arrest: propensity-adjusted analysis. J Intensive Care Med.

[5] Dumas F, Cariou A, Manzo-Silberman S, Grimaldi D, Vivien B, Rosencher J (2010). Immediate percutaneous coronary intervention is associated with better survival after out-of-hospital cardiac arrest: insights from the PROCAT (Parisian Region Out of hospital Cardiac ArresT) registry. Circ Cardiovasc Interv.

[6] Neumar RW, Nolan JP, Adrie C, Aibiki M, Berg RA, Bottiger BW (2008). Post-cardiac arrest syndrome: epidemiology, pathophysiology, treatment, and prognostication. A consensus statement from the International Liaison Committee on Resuscitation (American Heart Association, Australian and New Zealand Council on Resuscitation, European Resuscitation Council, Heart and Stroke Foundation of Canada, InterAmerican Heart Foundation, Resuscitation Council of Asia, and the Resuscitation Council of Southern Africa); the American Heart Association Emergency Cardiovascular Care Committee; the Council on Cardiovascular Surgery and Anesthesia; the Council on Cardiopulmonary, Perioperative, and Critical Care; the Council on Clinical Cardiology; and the Stroke Council. Circulation.

[7] Nielsen N, Hovdenes J, Nilsson F, Rubertsson S, Stammet P, Sunde K (2009). Outcome, timing and adverse events in therapeutic hypothermia after out-of-hospital cardiac arrest. Acta Anaesthesiol Scand.

[8] Ogawa T, Akahane M, Koike S, Tanabe S, Mizoguchi T, Imamura T (2011). Outcomes of chest compression only CPR versus conventional CPR conducted by lay people in patients with out of hospital cardiopulmonary arrest witnessed by bystanders: nationwide population based observational study. BMJ.

[9] Iwami T, Kawamura T, Hiraide A, Berg RA, Hayashi Y, Nishiuchi T (2007). Effectiveness of bystander-initiated cardiac-only resuscitation for patients with out-of-hospital cardiac arrest. Circulation.

[10] Sideris G, Voicu S, Dillinger JG, Stratiev V, Logeart D, Broche C (2011). Value of post-resuscitation electrocardiogram in the diagnosis of acute myocardial infarction in out-of-hospital cardiac arrest patients. Resuscitation.

[11] Aurore A, Jabre P, Liot P, Margenet A, Lecarpentier E, Combes X (2011). Predictive factors for positive coronary angiography in out-of-hospital cardiac arrest patients. Eur J Emerg Med.

[12] Garot P, Lefevre T, Eltchaninoff H, Morice MC, Tamion F, Abry B (2007). Six-month outcome of emergency percutaneous coronary intervention in resuscitated patients after cardiac arrest complicating ST-elevation myocardial infarction. Circulation.

[13] Hosmane VR, Mustafa NG, Reddy VK, Reese CL, DiSabatino A, Kolm P (2009). Survival and neurologic recovery in patients with ST-segment elevation myocardial infarction resuscitated from cardiac arrest. J Am Coll Cardiol.

[14] McMullan PW, White CJ (2010). Doing what’s right for the resuscitated. Catheter Cardiovasc Interv.

[15] Lindenauer PK, Remus D, Roman S, Rothberg MB, Benjamin EM, Ma A (2007). Public reporting and pay for performance in hospital quality improvement. N Engl J Med.

[16] Wang CH, Kuo LT, Hung MJ, Cherng WJ (2002). Coronary vasospasm as a possible cause of elevated cardiac troponin I in patients with acute coronary syndrome and insignificant coronary artery disease. Am Heart J.

[17] Rigatelli G, Rigatelli G, Rossi P, Docali G (2004). Normal angiogram in acute coronary syndromes: the underestimated role of alternative substrates of myocardial ischemia. Int J Cardiovasc Imaging.

